# Nearly Perfect Durable Superhydrophobic Surfaces Fabricated by a Simple One-Step Plasma Treatment

**DOI:** 10.1038/s41598-017-02108-1

**Published:** 2017-05-16

**Authors:** Jeongeun Ryu, Kiwoong Kim, JooYoung Park, Bae Geun Hwang, YoungChul Ko, HyunJoo Kim, JeongSu Han, EungRyeol Seo, YongJong Park, Sang Joon Lee

**Affiliations:** 1 Center for Biofluid and Biomimic Research, Department of Mechanical Engineering, Pohang University of Science and Technology, Pohang, 790-784 Republic of Korea; 2 Digital Appliance Advanced R&D Team, Samsung Electronics, Suwon, 443-742 Republic of Korea

## Abstract

Fabrication of superhydrophobic surfaces is an area of great interest because it can be applicable to various engineering fields. A simple, safe and inexpensive fabrication process is required to fabricate applicable superhydrophobic surfaces. In this study, we developed a facile fabrication method of nearly perfect superhydrophobic surfaces through plasma treatment with argon and oxygen gases. A polytetrafluoroethylene (PTFE) sheet was selected as a substrate material. We optimized the fabrication parameters to produce superhydrophobic surfaces of superior performance using the Taguchi method. The contact angle of the pristine PTFE surface is approximately 111.0° ± 2.4°, with a sliding angle of 12.3° ± 6.4°. After the plasma treatment, nano-sized spherical tips, which looked like crown-structures, were created. This PTFE sheet exhibits the maximum contact angle of 178.9°, with a sliding angle less than 1°. As a result, this superhydrophobic surface requires a small external force to detach water droplets dripped on the surface. The contact angle of the fabricated superhydrophobic surface is almost retained, even after performing an air-aging test for 80 days and a droplet impacting test for 6 h. This fabrication method can provide superb superhydrophobic surface using simple one-step plasma etching.

## Introduction

The wettability of a solid surface is important in a wide range of academic science and engineering applications. As an extreme state of surface wettability, superhydrophobicity has received considerable attention because of its strong potential in various fields including self-cleaning^1, 2^, anti-fogging^3, 4^, anti-frosting^5–10^, anti-icing^11–13^, condensed microdrop self-removal^14–19^, water collection^20, 21^ and enhancing condensation heat transfer^22–25^. Various fabrication methods of superhydrophobic surfaces have been introduced in the past few years, including the growth and synthesis of nanostructures^26, 27^, coating of nanoparticles or nanofilaments^28, 29^, wet-etching with chemicals^30–33^, and dry-etching with SF_6_ and CHF_3_ gases^34^. Despite technical advances in fabrication methods of superhydrophobic surfaces, many of these methods require multi-step processes^29, 30^ and post-chemical treatments^27, 35^. Thus, the development of a simple, cost-effective and environmentally friendly fabrication method of superhydrophobic surface is strongly and timely required.

Plasma etching treatment has been proven as a simple method for modifying the surface properties. Surface structure is modified by the bombardment of excited ions, which are generated from the plasma, to the substrate^36^. This fabrication method can alter the wettability of substrate by increasing roughness^37^ or changing functional group of the surface^38^. When the plasma was treated with only argon gas on a polytetrafluoroethylene (PTFE) substrate, the surface attained hydrophilic property due to formation of the peroxy radical bond to carbon as the cross-linked structure^39^. The hydrophobic surface could be obtained by applying reactive plasma generated from argon and oxygen mixture on the PTFE substrate^36, 40^. The surface morphology was changed to have microstructures with various surface roughness. However, they did not mention the sliding behavior of water droplets on the fabricated surface. In addition, formation of microstructures was studied only against treatment time. Thus, a systematic investigation on the important parameters of plasma treatment is strongly required for better understanding about the relationship between treatment parameters and surface wettability.

In this study, we propose a simple one-step plasma treatment method for fabricating superb superhydrophobic surface on a fluorocarbon-based polymer, which has a low surface energy of 20 mN m^−1^ at 20 °C. We optimize the fabrication parameters of superhydrophobic surfaces through plasma treatment with argon and oxygen gases by adopting the Taguchi method. The performance of the fabricated surfaces is examined by measuring their wetting properties, such as static contact angle, sliding angle, and self-cleaning effect. In addition, we demonstrate superior stability in the superhydrophobicity of the fabricated PTFE surface.

## Results

### Fabrication of optimized superhydrophobic PTFE sheets

To fabricate superb superhydrophobic surface, parameters of plasma etching should be optimized. As the first step, argon and oxygen gases were utilized in the plasma treatment (Figure S1) because they are inexpensive and safe in terms of chemical reactions. The important parameters in the plasma treatment are the total amount of gas, the gas flow rate ratio of argon to oxygen gases, the RF power, and the plasma exposure time to fabricate superhydrophobic PTFE sheets. We conducted an optimization analysis of the fabrication of superhydrophobic PTFE sheets (Supporting Information). Based on the Taguchi method, RF power was found to be the most influential factor, and plasma exposure time was the second important factor (Figure S2). The total amount of gas and the flow rate ratio of argon to oxygen gases were less influential. In this optimization analysis, the best fabrication condition was obtained at a total gas flow of 16 sccm and 5:3 ratio of argon and oxygen gases for a 3 h plasma treatment. The effect of RF power on the fabrication of superhydrophobic PTFE surfaces was analyzed by further dividing into finer power levels, and the optimized RF power was 150 W (Figure S3).

**Figure 1:**
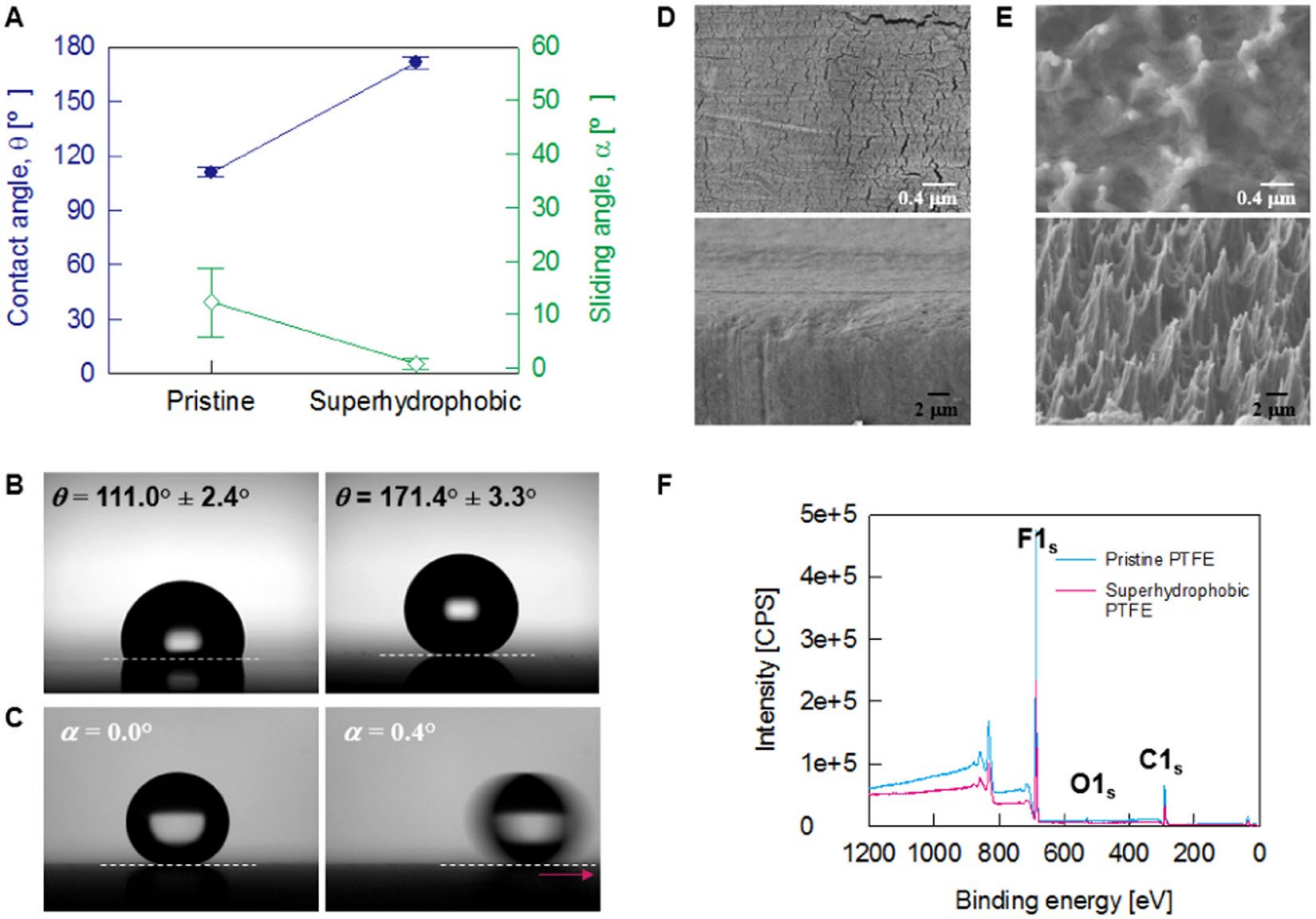
Wetting behaviors and surface characteristics of the fabricated superhydrophobic PTFE sheets. (**A**) Variations of the contact angle (marked by blue circle) and the sliding angle (marked by green diamond) of the pristine PTFE and the fabricated superhydrophobic PTFE sheets. (**B**) Contact-angle images of the pristine PTFE sheet (left) and the superhydrophobic PTFE sheet (right). (**C**) Sliding angle images of the superhydrophobic PTFE sheet. Water droplet disappears when the inclined angle is approximately 0.4°. SEM images of (**D**) the pristine PTFE having a smooth surface and (**E**) the fabricated superhydrophobic PTFE having crown-shaped bumps. The top images are top-view images, and the bottom images are side-view images. (**F**) Broad spectra obtained by XPS analysis on the pristine PTFE sheets and the fabricated superhydrophobic PTFE sheets.

Using the optimized plasma treatment conditions, we could fabricate nearly perfect superhydrophobic PTFE surfaces (Fig. 1). The contact angle of the pristine PTFE surface was 111.0° ± 2.4°, with a sliding angle of 12.3° ± 6.4°. However, after the plasma treatment, the water contact angles of the optimized superhydrophobic PTFE sheets are larger than 170°, and the sliding angles are less than 1° (Fig. 1A and B). Figure 1C shows consecutive images revealing the movement of a water droplet with the volume of 4.8 µL on the fabricated superhydrophobic PTFE surface tilted from 0° to 0.4°. The static contact angle is approximately 171.4° ± 3.3°. When the tilting angle (α) becomes 0.4°, the water droplet starts to slide off the surface. At the sliding angle of 0.4°, the contact angle hysteresis of the fabricated superhydrophobic PTFE surface is estimated to be 1~2°.

On the surface of the fabricated superhydrophobic PTFE sheets, nano-scale sharp protrusions were compactly formed, and a sphere was especially formed at the tip of the protrusion (Fig. 1E). These spheric structures may be the key for producing excellent superhydrophobicity (Figure S4). The SEM image of the side view shows that the superhydrophobic PTFE surface has uniformly distributed nano-sized pointed projections of several hundred nanometers in physical dimension. While the surface roughness of the pristine PTFE surface before the surface treatment was almost 111 nm (Figure S5A), the surface roughness was increased to 342 nm after the plasma treatment (Figure S5B).

The plasma treatment using argon and oxygen gases appear to physically change the surface structure and provide superhydrophobicity without changing chemical properties of the target surface. From the XPS analysis results, the PTFE sheets before and after the plasma treatment exhibit no change in chemical properties (Fig. 1F). In the broad spectrum of XPS, the energy regions of the major elements C, O, and F are not significantly changed (Table 1). The peak positions for the pristine and plasma-treated superhydrophobic PTFE sheets are found to coincide within 0.5 eV in the narrow-scan XPS spectra for C1s and F1s species (Figure S6). These results show no chemical changes of PTFE surface after plasma treatment.

**Table 1:** Elemental composition of the pristine and plasma-treated superhydrophobic PTFE sheets from XPS broad spectra.

Component (at%)	C	O	F
Pristine PTFE	34.45	1.08	64.47
Superhydrophobic PTFE	33.85	1.9	64.25

### Wetting characteristics of the fabricated superhydrophobic PTFE sheets

**Figure 2:**
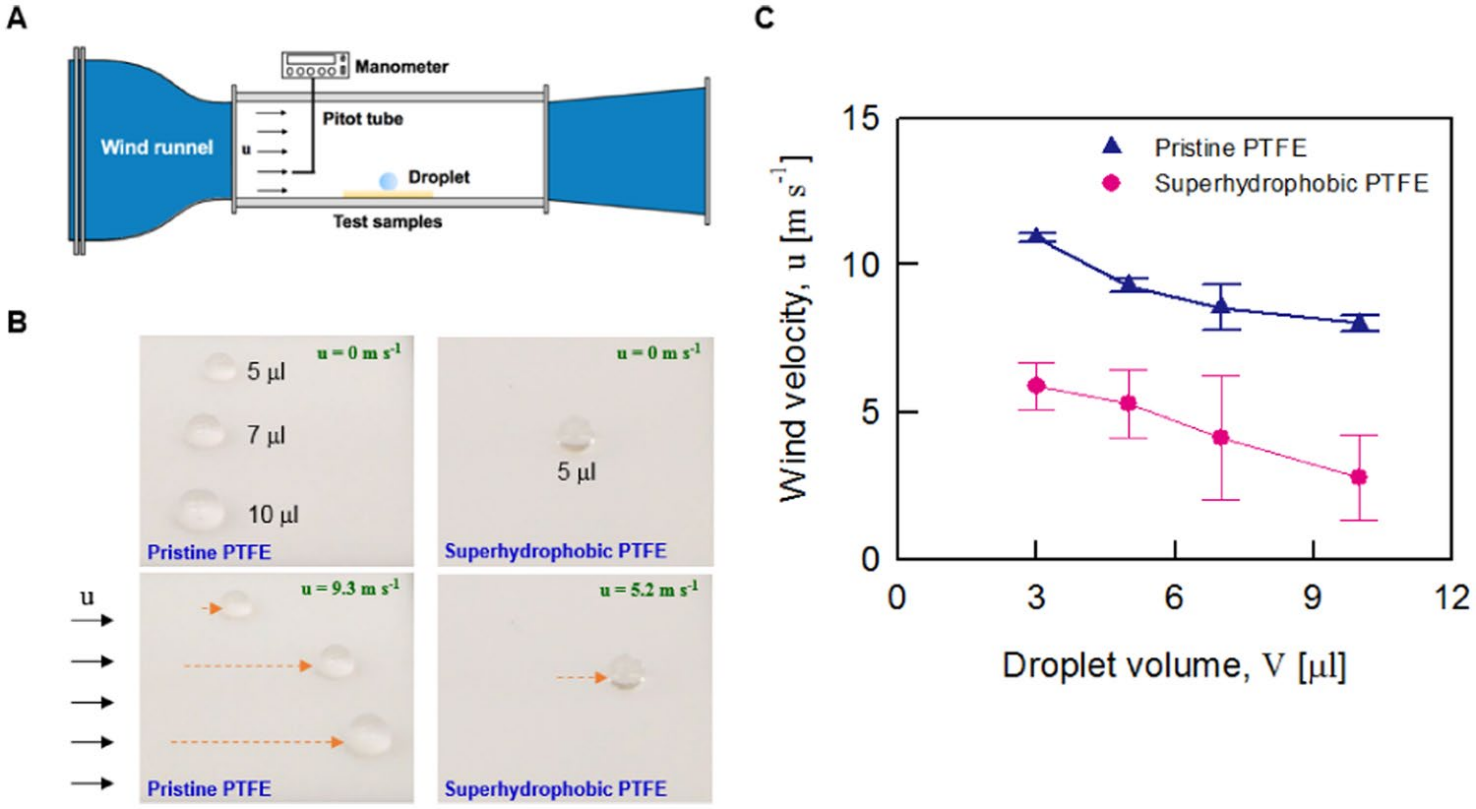
Self-cleaning effect of the fabricated superhydrophobic PTFE sheets. (**A**) The removal of water droplets of 5 μL, 7 μL, and 10 μL in volume was examined with varying wind speed. The test samples were attached on the bottom of the wind tunnel test section. Wind velocity was measured by a Pitot tube. (**B**) Water droplets on the pristine PTFE start to move when the wind velocity is 9.3 m s^−1^. A water droplet of 5 μL in volume starts to move at 5.2 m s^−1^. (**C**) Variations of the wind velocity to detach various volumes of water droplets attached on the pristine PTFE and superhydrophobic PTFE sheets.

We demonstrated the wetting characteristics of the fabricated superhydrophobic PTFE sheets *via* a water drop detachment experiment with changing wind speed in a small wind tunnel (Fig. 2A). Droplets that were 3 µl in volume started to move on the hydrophobic pristine PTFE sheets at a wind speed of 10.9 m s^−1^. Water droplets that were 5 µl, 7 µl, and 10 µl in volume started to move at a wind speed of 9.3 m s^−1^, 8.5 m s^−1^, and 8.0 m s^−1^, respectively (Fig. 2B and C). In the superhydrophobic PTFE surfaces, water droplets that were 3 µl, 5 µl, 7 µl, and 10 µl in volume were completely removed from the surface, even at low wind speeds in the range from 2.8 to 5.9 m s^−1^.

These results exhibited that smaller droplets needed a larger wind speed to move on the surface. In this situation, droplets can be detached from the surface by air flow when its drag force (*F*_*drag*_) exceeds the adhesion force (*F*_*adv*_). The drag force (*F*_*drag*_) created by the air flow with a free-stream velocity ($U$) is expressed using drag coefficient (*C*_*D*_) as follow: ${F}_{drag}=\frac{1}{2}\rho {U}^{2}A{C}_{D}$, where *ρ* is the density of air, and *A* is the frontal area of the water droplet. The adhesion force (*F*_*adh*_) of the droplet on the superhydrophobic PTFE surface can be controlled by varying the wetting parameters such as surface tension, contact angle and contact line. Assuming that the variation of contact angles along the contact line and the deviation of the contact line from a circular shape are negligible, the force can be expressed as the following simplified form, ${F}_{adh}=\gamma {L}_{b}(\cos \,{\theta }_{{\rm{\min }}}-\,\cos \,{\theta }_{{\rm{\max }}})$, where *γ* is the surface tension of water, *L*_*b*_ is the base length of the water droplet, and *θ*_min_ and *θ*_max_ are the minimum and maximum contact angles at upstream and downstream locations, respectively. Considering the force balance between *F*_*drag*_ and *F*_*adh*_, the critical wind velocity (*U*_*c*_) at which water droplets are blown off can be derived as follow: ${U}_{c}=\sqrt{2\gamma {L}_{b}(\cos \,{\theta }_{\min }-\,\cos \,{\theta }_{\max })/\rho A{C}_{D}}$. Since the term $\gamma (\cos {\theta }_{min}-\cos {\theta }_{max})$ is constant under the same wettability conditions, ${U}_{c} \sim \sqrt{{L}_{b}/A{C}_{D}}$. This indicated that a larger critical wind velocity is expected for smaller droplets. The force analysis on droplet detachment supports our experimental results showing that at the same wind speed, smaller water droplets move easily on the surface. The increasing trend of the critical wind velocity with decreasing the volume of liquid droplets is also matched with other previous studies^41, 42^.

In addition, the minimum wind speed at which water droplets started to move was much smaller on the superhydrophobic PTFE sheets than that on the pristine PTFE sheet. These results show that the fabricated superhydrophobic surfaces have lower adhesion force and better self-cleaning effect compared to the pristine PTFE sheets.

To demonstrate the self-cleaning effect of the proposed superhydrophobic surface, a dust removal test was conducted. Carbon nanopowders, of which size is ranged from a few microns to a few hundred microns, were used as dusts. As shown in Figure S7, the carbon dusts were sprayed onto the pristine PTFE and superhydrophobic PTFE. After the deposition of the sprayed dust particles, the surface was tilted to 15°. At this tilt angle of 15°, water droplets with a volume of 33.4 µL were dripped onto the surfaces. Water droplets were rolled down with sweeping the carbon nanopowders on the fabricated superhydrophobic PTFE surface (Movie S1). However, they were remained on the pristine PTFE sheet. After repeating the dripping of water droplets on the surfaces, the carbon nanoparticles were clearly removed from the fabricated superhydrophobic surface, whereas the dusts were remained on the pristine PTFE surface with water droplets. This result demonstrates that the fabricated superhydrophobic PTFE surface has self-cleaning effect as found from a lotus leaf in nature.

### Maintenance of superhydrophobic stability of plasma-treated PTFE sheets

**Figure 3:**
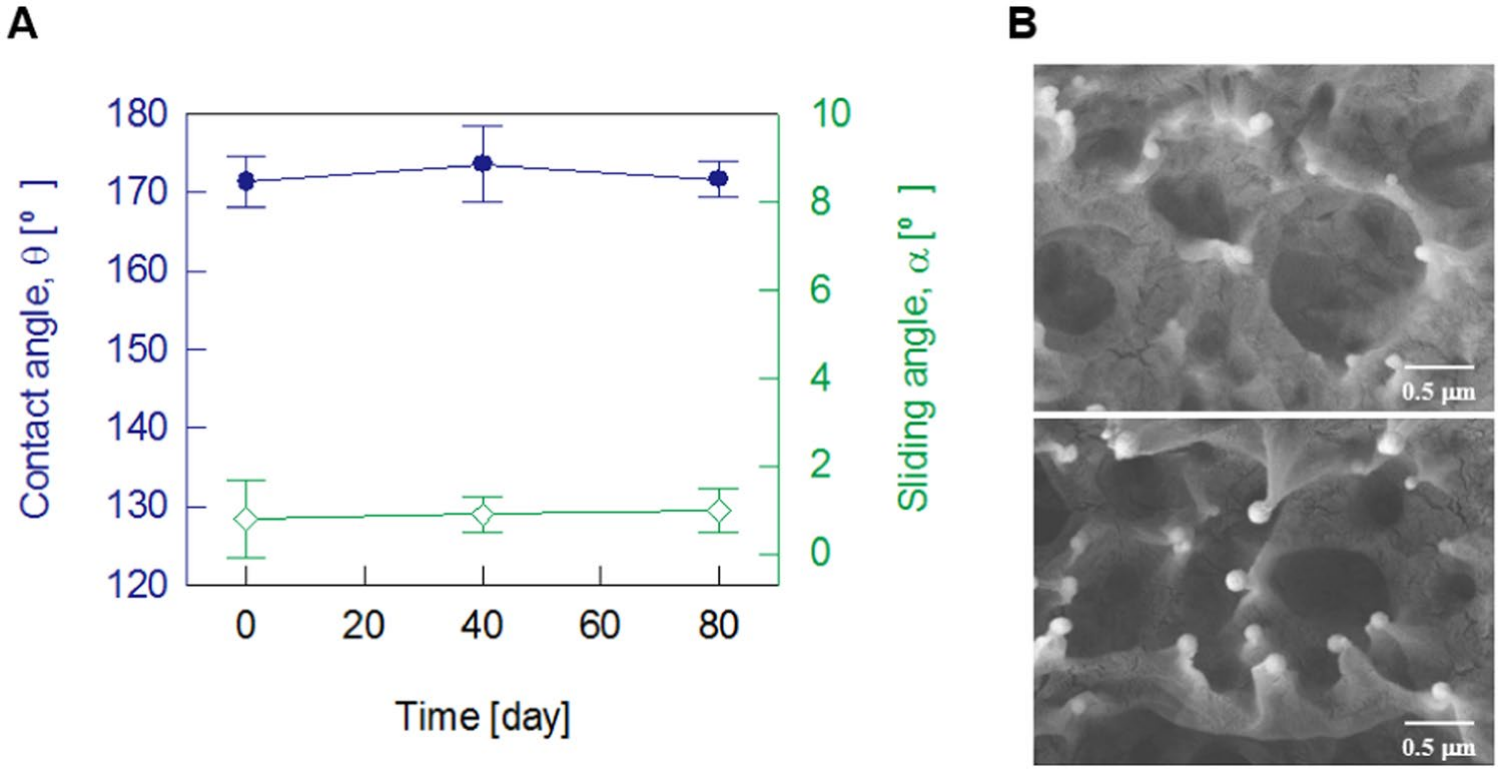
Surface durability of the fabricated PTFE sheets exposed to air for a long time. (**A**) The contact angle and the sliding angle of the superhydrophobic PTFE sheets (n = 6) are almost maintained after 80 days of air-aging. (**B**) SEM images before the aging test (top) and after 80 days of exposure to air (bottom). The crown-shape bumps are almost maintained.

The fabricated superhydrophobic PTFE sheets were exposed to air for approximately 80 days to demonstrate the durability of their superhydrophobicity. The static contact angles and the sliding angles were measured at 40 days and 80 days after the surface modification and then compared with those of the freshly fabricated superhydrophobic PTFE sheets (Fig. 3A). The contact angle and sliding angle of the superhydrophobic PTFE sheets just after the surface treatment were almost maintained, despite being exposed to air for 80 days from the surface treatment. Even after 80 days of plasma treatment, a large contact angle of approximately 170° was maintained, and the sliding angle was less than approximately 1° (n = 6). A SEM image shows that the microstructures of the surface exposed to the air for a long time, especially the spherical structure formed at the tip of the projections, were maintained (Fig. 3B). This finding demonstrates that the superhydrophobicity of the fabricated superhydrophobic sheet is unaffected, even after long-term storage in atmosphere.

**Figure 4:**
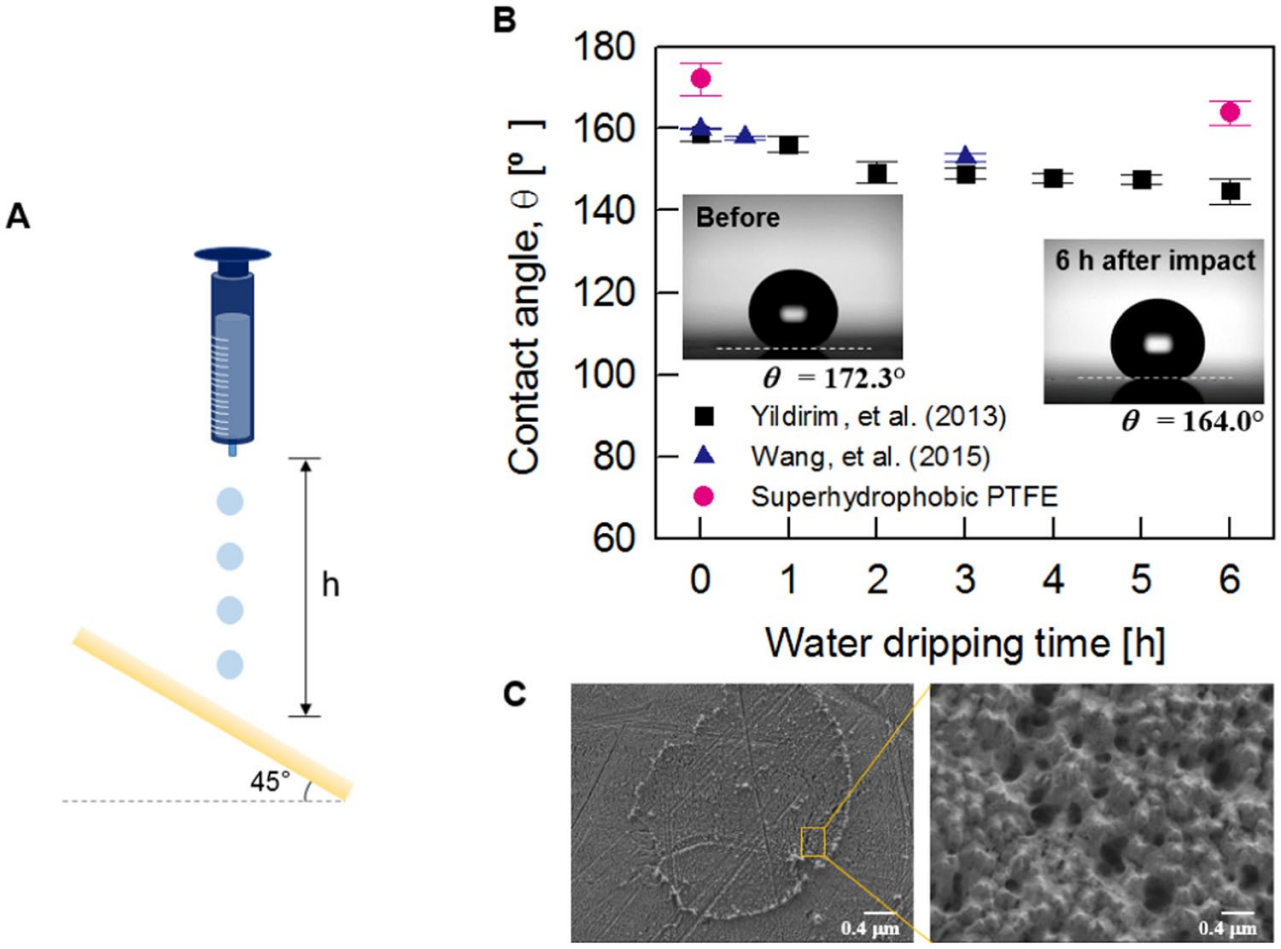
Stability of superhydrophobicity of the fabricated PTFE sheets as a function of excessive water dripping time. (**A**) In the water dripping test, water droplets (~8.6 μL) impacted the PTFE inclined at 45° from h = 10 cm at a rate of one drop per second. (**B**) Variation of the water contact angle of the superhydrophobic surfaces with the lapse of time for 6 h of water dripping of Yildirim, *et al*. (marked by black square), Wang, *et al*. (marked by blue upper-triangle), and the present superhydrophobic PTFE (marked by red circle). The contact angle of the fabricated superhydrophobic PTFE is slightly decreased, similar to that of the other results. (**C**) SEM images of the superhydrophobic surfaces after the water dripping test. The mark of droplets impacting on the surfaces was remained at the specific point where the water droplet impacted (left). The enlarged image shows that the nanostructures of the initial superhydrophobic surface are almost maintained (right).

The water droplet impact test was conducted on the fabricated superhydrophobic sheet to simply check the basic mechanical durability. In the water dripping test, an injection needle (23 G) was located 10 cm above the fabricated superhydrophobic surface, which was tilted by 45° (Fig. 4A). Water droplets that were 8.6 µl in volume were dripped onto the surface at a rate of one drop per second.

After exposing the fabricated superhydrophobic surface to water droplets for 6 h, the changes in the contact angle and surface morphology were examined. Water droplets were perfectly bounced off the fabricated surface during the whole durability test (Movie S2). After 6 h (~21,600 water drops) of the water dripping test, the superhydrophobicity of the fabricated surface was retained, although the static contact angle was slightly decreased from 172.3° to 164.0° (Fig. 4B). The superhydrophobic PTFE sheets fabricated in this study are superior to the previous superhydrophobic surfaces in terms of maintenance of high contact angle^43–45^. Figure 4C shows typical SEM images of the surfaces after the water dripping test. A round-shaped mark was left at the point where the water droplet impinged continuously, because PTFE surface has high wear rate under cyclic loading^46^. The magnified SEM image shows that the original nanoscale structure of hundreds of nanometers were partially crushed by repetitive impact of water droplets. The slight deformation of the fabricated surface seems to induce small decrease in contact angle. However, the proposed superhydrophobic surface exhibits not only the best contact angle but also superior superbydrophobicity even after the durability test. This result indicates that the fabricated superhydrophobic PTFE surface is quite durable, even after repetitive water drop impacts.

## Discussion

In this study, nearly perfect superhydrophobic PTFE specimens with a high contact angle up to 178° and an extremely low sliding angle less than 1° were fabricated *via* a simple one-step plasma treatment using only argon and oxygen, both of which are inexpensive and safe reactive gases. The fabricated superhydrophobic surfaces exhibit excellent superhydrophobicity in the aging test; they almost maintain the initial wettability, even after prolonged exposure to air for 80 days, and exhibit good water repellency with maintaining a high contact angle, even after the repetitive water droplet impact experiments. This fabrication method can be equally applicable to a thin film of PTFE. We believe that the superhydrophobic surface fabricated *via* plasma etching method using safe gases can be a good candidate to develop tip-like polymer nanoarrays with special performances in a simple process.

## Methods

### Materials

A commercial polytetrafluoroethylene (PTFE) sheet manufactured by a chemical company (Hyunwoo Chemical, Korea) was selected as the material for surface modification *via* plasma treatment. As a fluorocarbon-based polymer, PTFE has a low surface free energy of 20 mN m^−1^ at 20 °C^47^, which permits easy fabrication of superhydrophobicity^48^. In addition, PTFE has high chemical resistance, low- and high-temperature capability, resistance to weathering, low friction, electrical and thermal insulation and non-adhesive properties. The flat PTFE sheet used in this study has a physical dimension size of 3 cm × 3 cm and a thickness of 2 mm. The PTFE sheets were washed with acetone and isopropanol (Sigma-Aldrich, Korea) and then dried using an air-gun.

### Surface modification *via* plasma treatment

The surface morphology of the PTFE sheet was modified *via* plasma etching using Ar and O_2_ gases (Figure S1). A clean pristine PTFE was loaded inside the vacuum chamber, and then the chamber was evacuated to the operating pressure of approximately 1.0 × 10^−1^ Torr. Surface modification was conducted using the excited plasma induced by radiofrequency at 13.56 MHz. The maximum RF power was 600 W. The flow rates of both gases delivered to the chamber were controlled by varying mass flow controllers up to 30 sccm, and the temperature in the chamber was monitored using a K-type thermocouple. Plasma treatment on polymer substrates can change the surface wettability of the specimen to be hydrophilic^49^, but we employed the plasma treatment to fabricate superhydrophobic surface.

Among various surface treatment methods used to fabricate a superhydrophobic surface, the plasma-based dry etching method, which requires the use of reactive gases, was employed in this study. This method does not require handling dangerous acids and solvents. In addition, the fabrication process is relatively clean and safe compared to the wet etching method. In this study, Ar and O_2_ gases were used to fabricate the proposed superhydrophobic surface instead of poisonous gases, such as CHF_3_ and SH_6_^50^. Ar and O_2_ gases are economical and environmentally friendly gases. To generate plasma, AC power which is suitable for plasma treatment uniformly on a target surface, was chosen instead of DC power which has good linearity.

### Analysis of the physical and chemical characteristics of the fabricated superhydrophobic surface

To investigate the wettability property of the fabricated surface, the contact angle and sliding angle of the pristine and modified sheets were measured by dripping a sessile deionized water droplet with a volume of 5 μl (SmartDrop, Femtofab, Korea). For analyzing the durability of the fabricated surface, the fabricated superhydrophobic PTFE sheets were placed in air for more than two months and were the surface wettability was consecutively checked.

The structural features of the test samples were analyzed using a high resolution FE-SEM (JEOL JSM-7401F, JEOL, Ltd., Japan). To avoid the charging effect on non-conducting surfaces, a platinum coating was deposited for 30 s. In addition, the surface roughness was examined by using an AFM/STM base SPM system, VEECO Dimension Nanoscope V (Version 7.0) (Veeco Instruments, USA). The images of samples surfaces over a field of 50 μm × 50 μm were acquired and analyzed with spatial resolution of 0.1 nm (X-Y) and 0.01 nm (Z).

The surface chemistry of the test samples was examined using an X-ray photoelectron spectroscope (XPS) (ESCALAB 250, Thermo Fisher Scientific, USA). The X-ray source operated at 15 kV and 10 mA exciting the sample surface with a monochromatic Al−K_α_ (*hν* = 1486.6 eV) X-ray beam, which emits photoelectrons from the sample surface. Using the binding energy and the intensity at the photoelectron peak, the elemental identity, chemical state, and quantity of the detected elements were determined.

To test the mechanical durability of the superhydrophobic surface by using the long-term drop impact method^51^, water droplets, 8.6 µl in volume, were impinged onto the plasma-treated PTFE sheets at a height of 10 cm above the sample surface. The impact velocity was set to 1.4 m s^−1^, with the one-droplet impact occurring in a second. The substrate was tilted by 45° to ensure the drain off of the water droplets.

### Detachment of droplets from the fabricated surface

The removal of water droplets on the fabricated superhydrophobic surface was tested using a wind tunnel. The test samples were attached inside the wind tunnel test section, and then deionized water droplets with various volume sizes were placed on the superhydrophobic surface. The wind velocity was controlled by using a frequency modulation device (LG industrial systems, Korea). A Pitot tube was installed inside the test section, and the velocity on the superhydrophobic surface was measured using a digital manometer.

## Electronic supplementary material

Supplementary Info

Movie S1

Movie S2
