# Supplementary material for: Nearly Perfect Durable Superhydrophobic Surfaces Fabricated by a Simple One-Step Plasma Treatment
Source: Sci Rep. 2017 May 16;7:1981. doi: 10.1038/s41598-017-02108-1 (PMC5434029; doi:10.1038/s41598-017-02108-1)
Supplement: Supplementary file 1 — Supplementary Info [file 41598_2017_2108_MOESM1_ESM.pdf]

Nearly Perfect Durable Superhydrophobic Surfaces Fabricated by a Simple One-Step Plasma Treatment

Jeongeun Ryu[1,*], Kiwoong Kim[1,*], JooYoung Park[1], Bae Geun Hwang[1], YoungChul Ko[2], HyunJoo Kim[2], JeongSu Han[2], EungRyeol Seo[2], YongJong Park[2], and Sang Joon Lee[1,¶]

Optimization process for fabricating superhydrophobic PTFE sheets. To determine the optimized conditions for the fabrication of a nearly perfect superhydrophobic surface, we set the total gas flow, the gas flow rate ratio ([Ar]:[O2]), the RF power, and the plasma exposure time as the main control parameters (Table S1). The total amount of gases used in the optimization process was set to 16 sccm, 32 sccm, and 48 sccm. The ratio of argon to oxygen gas ([Ar]:[O2]) was set to 5:1, 5:3, and 5:5. The temperature inside the plasma chamber increases with RF power and plasma exposure time. As a result, the maximum temperature inside the chamber was adjusted so that it would not exceed 200 ℃. RF power was applied at 50 W, 100 W, and 200 W, and the plasma exposure time was set to 20 minutes, 60 minutes, and 180 minutes.

We constructed a $L_9(3^4)$ orthogonal array table composed of 9 experimental combinations of four main control parameters with different levels based on the Taguchi method (Table S2). When the total amount of gas was 16 sccm, the ratio of argon and oxygen gas was 5:3, the RF power of 100 W was applied for 60 minutes (P2), and the average contact angle was larger than 150°. When the total amount of gas was doubled to 32 sccm, the ratio of argon to oxygen gas was controlled to 5:1 and 5:3, and the RF power was applied at 100 W and

200 W in each run for 180 minutes and 20 minutes (P4 and P5, respectively); a surface having an average contact angle of 150° or more was also obtained. The average sliding angle of the pristine PTFE surface before plasma treatment was larger than 10°. However, the average sliding angle decreased to approximately 6° for the surface with a large contact angle (P2 and P3), and a small sliding angle within 1° was obtained.

Figure S2 shows the results of analyzing the effect of each factor on the fabrication of a superhydrophobic surface from the experiments on the 4 main factors, which were based on the Taguchi method. By comparing the S/N ratios of the parameters by the level of the factor, the RF power had the largest effect on the contact angles between the levels, and the plasma exposure time had the next highest level difference. The total amount of gas or the ratio of argon to oxygen gas showed little difference between the levels, and the difference between the two factors was not large. The results of the Taguchi method showed that the best contact angle could be obtained at 16 sccm and 5:3 ratio of argon and oxygen gas. The results also showed that the contact angles could be increased with the plasma exposure time.

The most important factor is RF power, which is further divided into finer RF power levels. All three parameters except RF power were controlled under the same conditions. The RF power value was set to 50 W, 70 W, 90 W, 100 W, 150 W, and 200 W. The test surface was modified under the plasma condition for 180 minutes. The contact angles were increased with RF power in a nonlinear sigmoidal fashion: $y = 107.5 + 58.5/(1 + exp\left(-\frac{x-68.2}{19.6}\right))$, R^2= 0.98 (Fig. S3). The dotted lines indicate the 95% confidence interval. When the RF power was larger than 100 W, a superhydrophobic surface with a surface contact angle of 150° or larger could be fabricated. In particular, at power conditions of 150 W and 200 W, the superhydrophobic wetting property of the surface was excellent enough to make it impossible

to fix the droplet with a volume of 5 μl on the fabricated superhydrophobic surface.

We investigated the effect of plasma exposure time on surface modification toward superhydrophobicity (Fig. S4). The contact angles increased with exposure time, and the sliding angles decreased with time (Fig. S4A). When exposure time was controlled to 60 min, the contact angle of target PTFE surfaces almost reached approximately 150°. However, the sliding angles of the fabricated PTFE surfaces were nearly the same as those of the pristine PTFE surfaces. After plasma treatment for 180 min, the contact angles exceeded 170°, and the sliding angles were less than 1°.

The change of surface wettability may be attributed to chamber temperature. Chamber temperature increased with plasma exposure time, and temperature was nearly approximately 150 ℃ (Fig. S4B). The surface morphology of the structures of the fabricated PTFE sheets after 1 h of exposure (Fig. S4C) showed nano-sized sharp protrusions around the hollow structure, which is similar to those of fabricated PTFE sheets after 3 h of exposure (Fig. S4D). However, short fiber structures were formed at the tip of the protrusion on the PTFE sheets under the plasma for 1 h. These structures may increase the adhesion force compared to the case of the best superhydrophobic PTFE sheets.

As a result of optimization of the plasma treatment process to obtain the superhydrophobic surface, the best superhydrophobic surface can be obtained with an RF power of 150 W, with the total amount of reactive gas controlled to 16 sccm, and the amounts of argon and oxygen gases adjusted to 10 sccm and 6 sccm, respectively. Plasma treatment for 3 hours under these conditions can produce the best superhydrophobic PTFE surface.

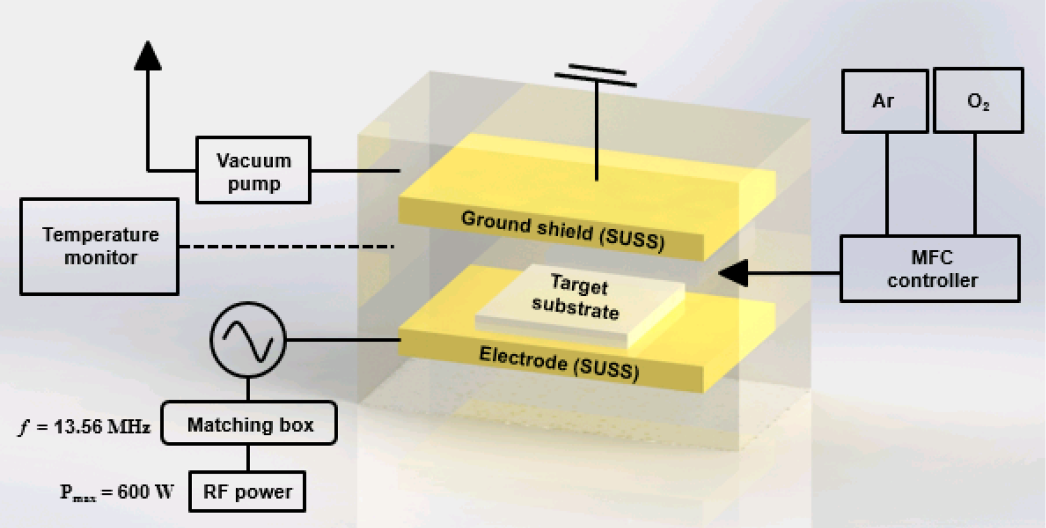

Figure S1. Schematic diagram of the Ar & O_2 plasma treatment. The pressure in the plasma treatment chamber was maintained at approximately 1.0×10^{-1} Torr using a vacuum pump, and the temperature was continuously monitored during the treatment process. The electrode was connected to the power generator with a maximum RF power of 600 W. The air influx was controlled by a mass flow controller (MFC). Target specimens were placed on the electrode. Surface modification was conducted using the excited plasma.

Table S1. Main parameters of plasma treatment with their different values used for the tests in this study.

Parameter designation	Variable	Variable level		
		Low	Central	High
A	Total gas flow (sccm)	16	32	48
B	Gas ratio, [Ar]:[O_2]	5 : 1	5 : 3	5 : 5
C	RF power (W)	50	100	200
D	Exposure time (min)	20	60	180

Table S2. Control factors used for optimization of the experimental conditions.

Run	Total gas flow (*A*)	Gas ratio [Ar]:[O_2] (*B*)	RF power (*C*)	Exposure time (*D*)
P1	16	5 : 1	50	20
P2	16	5 : 3	100	60
P3	16	5 : 5	200	180
P4	32	5 : 1	100	180
P5	32	5 : 3	200	20
P6	32	5 : 5	50	60
P7	48	5 : 1	200	60
P8	48	5 : 3	50	180
P9	48	5 : 5	100	20

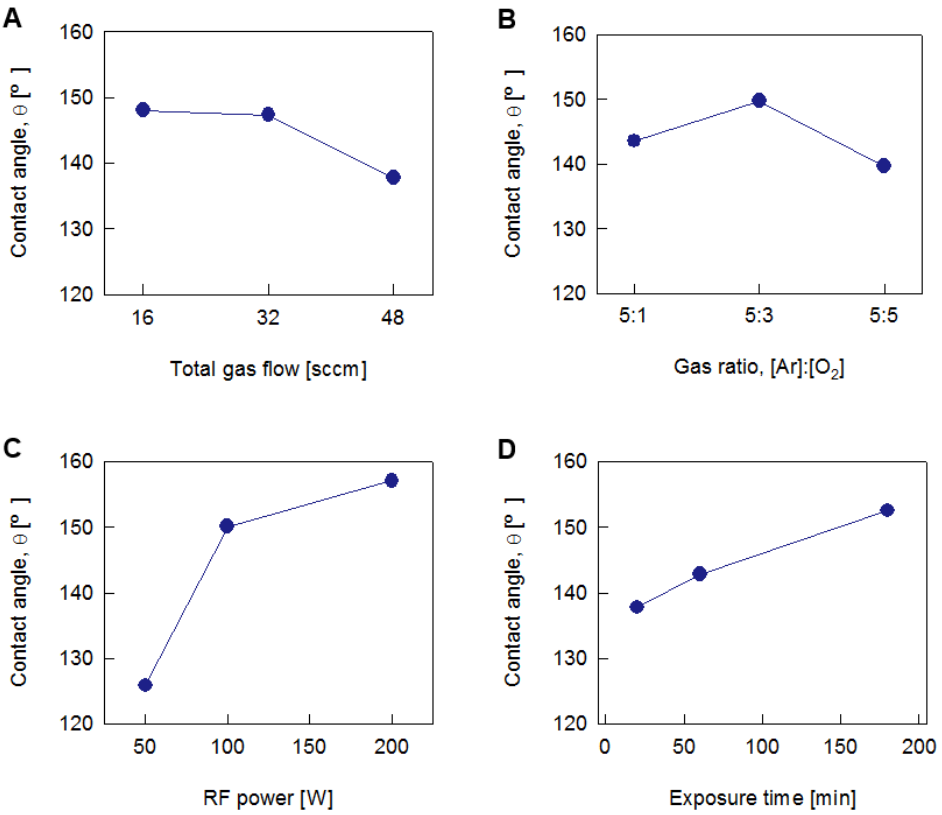

Figure S2. Effect of four parameters on the contact angle of the fabricated SHP PTFE sheets: (A) total gas flow, (B) gas ratio, (C) RF power, and (D) exposure time.

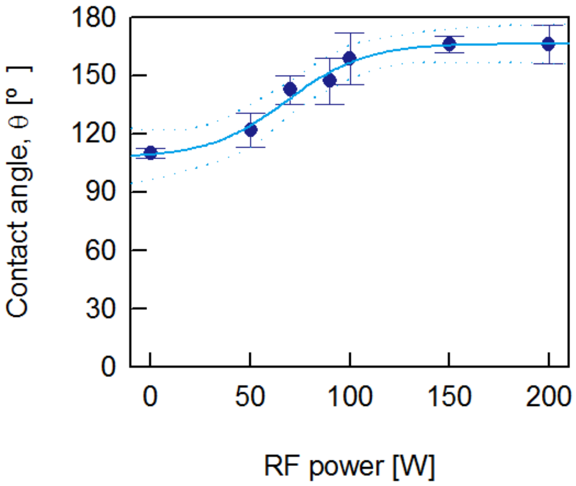

Figure S3. Effect of RF power on the contact angle of the fabricated SHP sheet at a fixed treatment time of 3 h.

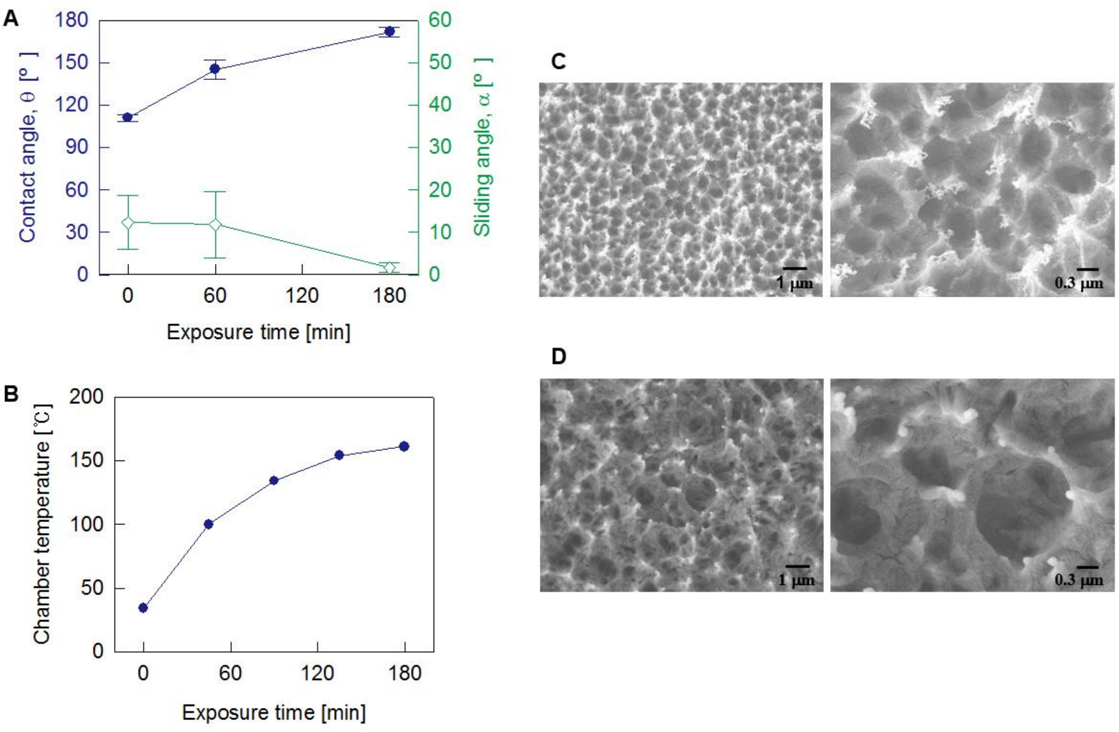

Figure S4. Effect of exposure time on the surface modification of the fabricated SHP sheet at a fixed RF power of 150 W. (A) Variations of the contact angle and the sliding angle according to exposure time. The contact angle increases and the sliding angle decreases as the exposure time increases. (B) Variations of the in-chamber temperature during the plasma treatment process. SEM images of (C) the 1-h treated PTFE sheet and (D) the 3-h treated PTFE sheet.

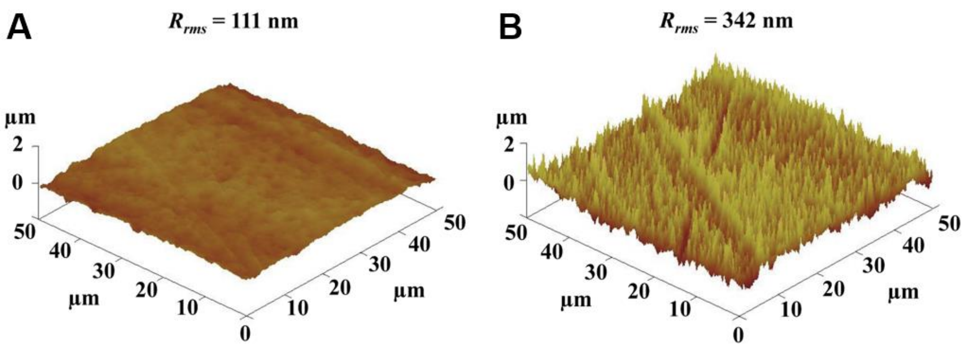

Figure S5. Surface characteristics of the fabricated superhydrophobic PTFE sheets. AFM images of (A) the pristine PTFE and (B) the superhydrophobic PTFE surfaces.

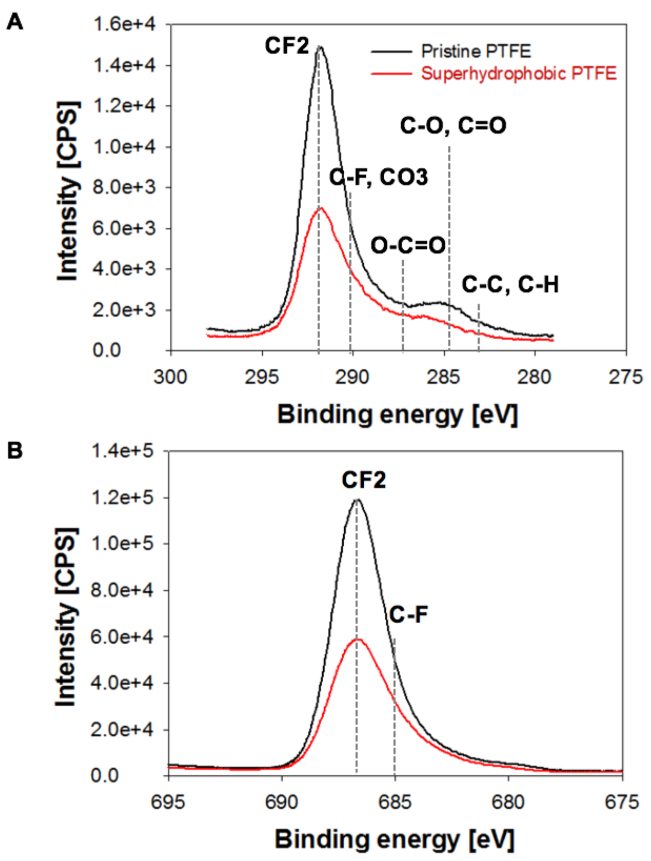

Figure S6. XPS high resolution (A) C1s spectra and (B) F1s spectra of the pristine and superhydrophobic PTFE films

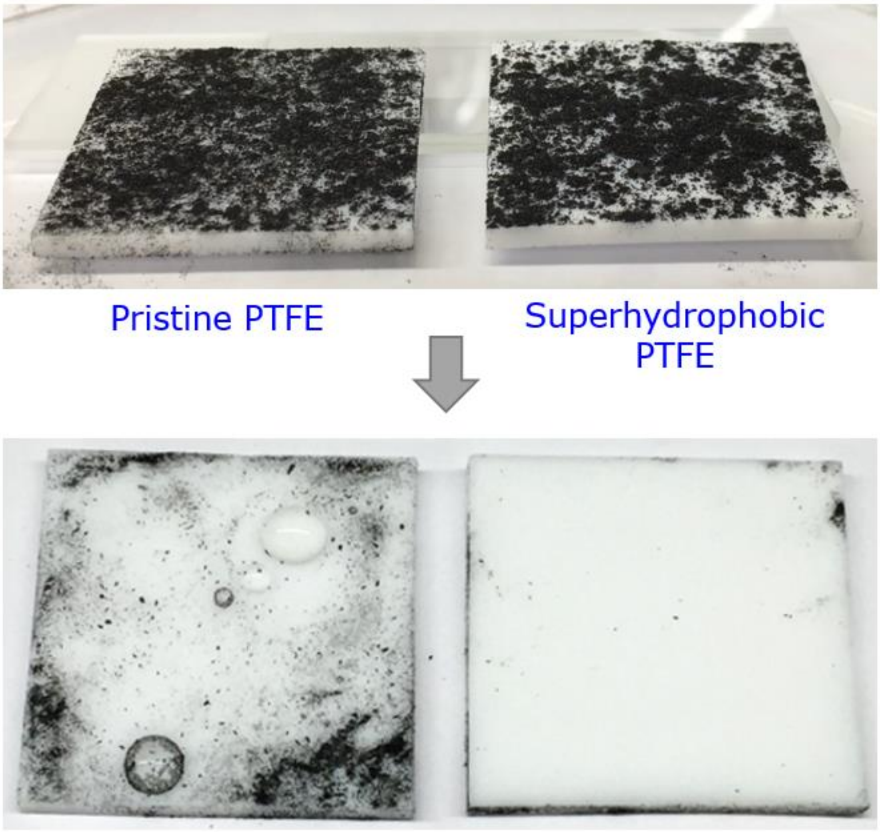

Figure S7. Demonstration of the self-cleaning effect of the fabricated superhydrophobic surface. The carbon nanopowders on the fabricated PTFE surface are almost removed by the rolling water droplets.

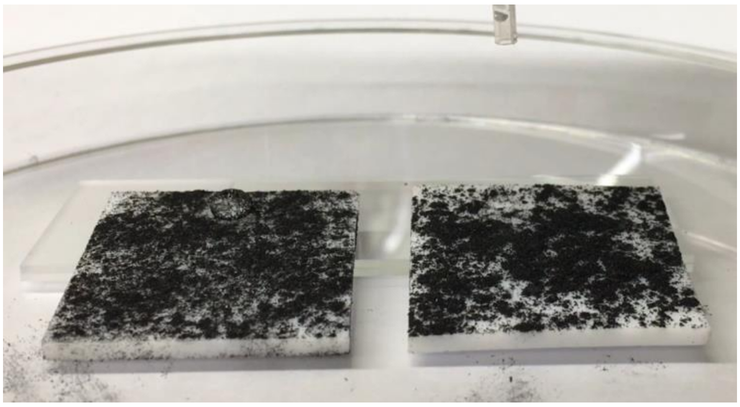

Movie S1. The water droplet rolled down with the carbon particles when it was applied on the fabricated superhydrophobic PTFE surface (right). However, the droplet was remained on the pristine PTFE with dust (left).

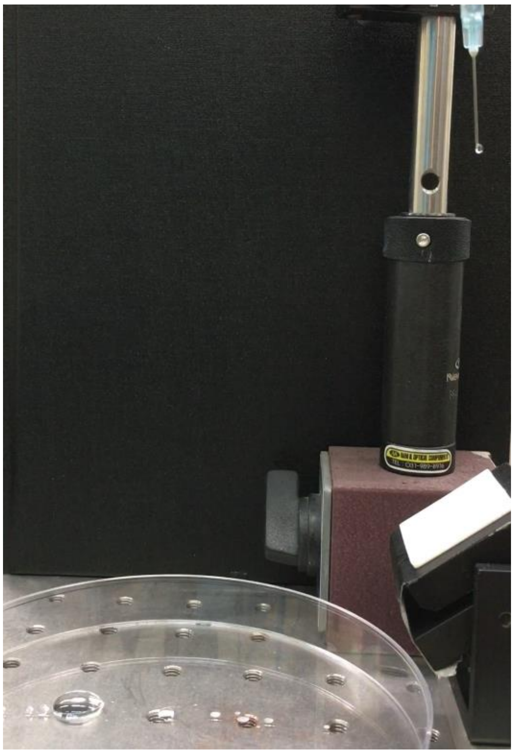

Movie S2. Water droplets were perfectly bounced off the fabricated surface during the whole durability test
